# A Logistic Regression Prediction Model for Post‐PCI In‐Hospital Major Adverse Cardiovascular Events in Patients With Acute ST‐Segment Elevation Myocardial Infarction: Construction and Internal Validation

**DOI:** 10.1155/cdr/3618662

**Published:** 2026-10-17

**Authors:** Jun Li, Geng Pan, Na Wang, Mei Wu

**Affiliations:** ^1^ Department of Cardiology Chongming Hospital Affiliated to Shanghai University of Medicine and Health Sciences Shanghai China sumhs.edu.cn; ^2^ Department of Interventional Radiology Room The Lu′an Hospital Affiliated to Anhui Medical University The Lu′an People′s Hospital Lu′an Anhui China; ^3^ Department of Gynaecology Chongming Hospital Affiliated to Shanghai University of Medicine and Health Sciences Shanghai China sumhs.edu.cn

**Keywords:** acute ST-segment elevation myocardial infarction, internal validation, LASSO regression, major adverse cardiovascular events, percutaneous coronary intervention, prediction model

## Abstract

**Background:**

The incidence of in‐hospital major adverse cardiovascular events (MACE) remains high in patients with acute ST‐segment elevation myocardial infarction (STEMI) after emergency percutaneous coronary intervention (PCI). Existing risk prediction tools mostly focus on preoperative baseline indicators and ignore intraoperative interventional parameters, lacking comprehensive postprocedural risk assessment tools for STEMI patients after PCI. This study is aimed at developing and internally validating a logistic regression prediction model for post‐PCI in‐hospital MACE in STEMI patients based on multidimensional perioperative clinical indicators, so as to realize accurate postoperative risk stratification and guide individualized perioperative management.

**Methods:**

We retrospectively included 1195 STEMI patients who underwent emergency PCI at the Chest Pain Center of Lu′an People′s Hospital from January 2019 to October 2024. Patients were randomly divided into training (*n* = 837) and validation (*n* = 358) sets at a 7:3 ratio. The primary endpoint was in‐hospital MACE (composite of cardiogenic shock, malignant arrhythmias, acute heart failure, reinfarction, or all‐cause death). A prediction model was constructed using least absolute shrinkage and selection operator (LASSO) regression combined with multivariate logistic regression. Model discrimination was evaluated via the area under the receiver operating characteristic curve (AUC). Head‐to‐head discriminative comparison with the widely used GRACE and TIMI risk scores was performed using AUC values and the DeLong test to quantify the incremental predictive value of the novel model. Model calibration and clinical utility were assessed via calibration plots, the Hosmer–Lemeshow test, and decision curve analysis (DCA), with internal validation performed in the validation set.

**Results:**

A total of 260 patients (21.8%) experienced in‐hospital MACE. After LASSO regression screening and multivariate logistic regression analysis, eight independent predictors were identified: admission systolic blood pressure, Killip classification, intervention duration, contrast volume, postprocedural TIMI flow grade, culprit vessel occlusion, serum creatinine, and serum potassium. The training set AUC was 0.84 (95% CI: 0.80–0.87) with a sensitivity of 77.7% and specificity of 78.2%; the validation set AUC was 0.826 (95% CI: 0.782–0.870). Comparative discriminative analysis showed that the traditional GRACE and TIMI risk scores exhibited moderate predictive performance. The training set AUC of the GRACE score was 0.763 (95% CI: 0.724–0.800), and that of the TIMI score was 0.748 (95% CI: 0.708–0.786). In the validation set, the AUC of the GRACE score and TIMI score were 0.751 (95% CI: 0.699–0.800) and 0.736 (95% CI: 0.683–0.786), respectively. The DeLong test confirmed that our novel model achieved significantly higher discriminative ability than both GRACE and TIMI scores in both training and validation cohorts (all *p* < 0.05). The training set Brier score was 0.128, with a calibration intercept of 0.042 and calibration slope of 0.991; the validation set Brier score was 0.135, with a calibration intercept of 0.051 and calibration slope of 0.985. After 1000 bootstrap optimism correction, the corrected calibration slope was 0.987 and the corrected intercept was 0.045. The Hosmer–Lemeshow test indicated good model calibration (*χ* ^2^ = 5.449, *p* = 0.709), and DCA demonstrated clinical net benefit within the 5%–80% threshold probability range.

**Conclusion:**

The logistic regression prediction model based on eight clinical indicators demonstrated good discrimination, quantitative calibration, and clinical utility for predicting post‐PCI in‐hospital MACE in STEMI patients. The model integrates preoperative baseline, intraoperative procedural, and laboratory indicators, achieves stable performance after internal validation, and can assist clinicians in postoperative risk stratification of patients, timely identifying high‐risk populations after PCI, and optimizing postoperative perioperative management strategies.

## 1. Introduction

Acute ST‐segment elevation myocardial infarction (STEMI) is a critical condition caused by acute complete coronary artery occlusion leading to extensive myocardial ischemia and necrosis. It is characterized by sudden onset, rapid progression, and high mortality, representing one of the leading causes of cardiovascular death globally [1, 2]. Percutaneous coronary intervention (PCI), as the preferred strategy for myocardial reperfusion in STEMI patients, has been recommended as the standard treatment by domestic and international clinical guidelines [3] and can significantly reduce both short‐ and long‐term mortality rates [4, 5].

However, even with timely PCI treatment, a considerable proportion of STEMI patients still experience major adverse cardiovascular events (MACE) during hospitalization, including cardiogenic shock, malignant arrhythmias, acute heart failure, reinfarction, and all‐cause death [6, 7]. In‐hospital MACE not only indicates poor prognosis but also significantly increases healthcare resource consumption [8]. Despite continuous improvements in chest pain center construction and advancing PCI techniques that have significantly shortened reperfusion times, the incidence of in‐hospital MACE remains high [9], suggesting that merely reducing reperfusion time is insufficient to comprehensively improve patient outcomes.

Accurate postprocedural risk stratification for STEMI patients after PCI is of significant clinical value for optimizing postoperative treatment strategies and rationally allocating medical resources. The GRACE and TIMI (thrombolysis in myocardial infarction) risk scores are the most widely applied classic risk stratification tools for STEMI patients in current clinical practice. Although existing risk assessment tools such as the GRACE score and TIMI risk score are widely used, they were primarily developed based on European and American populations, have certain limitations in predicting post‐PCI in‐hospital MACE in Chinese populations [10, 11], and only focus on patients′ preoperative clinical characteristics, ignoring key intraoperative interventional parameters closely related to prognosis (such as procedure duration and contrast volume), which cannot fully reflect the perioperative risk level of patients. Lacking intraoperative procedural indicators, these traditional scoring systems may underestimate or fail to capture postoperative risk alterations after PCI reperfusion, resulting in limited discriminative accuracy for postprocedural MACE prediction.

Therefore, based on single‐center clinical data from the Chinese chest pain center system, this study systematically integrated admission baseline characteristics, cardiac function assessment, intraoperative interventional parameters, and laboratory examination indicators. Using LASSO (least absolute shrinkage and selection operator) variable selection combined with multivariate logistic regression, we constructed and internally validated a comprehensive postprocedural prediction model for in‐hospital MACE after emergency PCI in STEMI patients. Furthermore, we conducted a head‐to‐head discriminative comparison with mainstream GRACE and TIMI risk scores to clarify the incremental predictive value of incorporating intraoperative perioperative indicators, aiming to provide a more accurate and practical tool for postoperative risk stratification, timely identification of post‐PCI high‐risk patients, and optimization of perioperative individualized management strategies.

## 2. Methods

### 2.1. Study Design and Ethical Statement

This was a single‐center retrospective cohort study. The study protocol was approved by the Ethics Committee of Lu′an People′s Hospital (Ethics Approval Number: 2024‐LY‐0278). Due to the retrospective nature of the study, informed consent was waived. This study adhered to the principles of the Declaration of Helsinki and was designed and written according to the TRIPOD (Transparent Reporting of a multivariable prediction model for Individual Prognosis Or Diagnosis) reporting guidelines [12].

### 2.2. Study Population

We consecutively included STEMI patients who presented to the Chest Pain Center of Lu′an People′s Hospital and received emergency PCI treatment from January 2019 to October 2024.

Inclusion criteria: age ≥ 18 years; first‐time acute myocardial infarction; STEMI diagnosis confirmed according to the 2017 European Society of Cardiology (ESC) STEMI management guidelines [13]; emergency PCI treatment after admission; hospital stay ≥ 5 days; and complete medical records. Exclusion criteria: previous myocardial infarction history; previous heart failure, cardiomyopathy, or severe valvular heart disease; severe liver or kidney dysfunction requiring artificial liver or hemodialysis treatment; history of psychiatric disorders; refusal of emergency PCI treatment or voluntary discharge with treatment abandonment; and missing key clinical data.

### 2.3. Data Collection

All patients had standardized personal files established upon admission, with clinical data collected and recorded by trained professional residents, including: admission baseline data: age, body mass index (BMI), admission systolic blood pressure (SBP), admission diastolic blood pressure (DBP), admission heart rate, Killip functional classification, gender, hypertension history, hyperlipidemia history, diabetes history, smoking history, and first medical contact (FMC) time. Interventional parameters: door‐to‐wire (D to W) time, intervention duration (from catheter sheath insertion to procedure completion), total contrast volume, preprocedural coronary TIMI flow grade, postprocedural TIMI flow grade, Gensini score, culprit vessel occlusion status, and culprit vessel location (left main, left anterior descending, left circumflex, and right coronary artery). Emergency laboratory indicators: white blood cell count, hemoglobin, platelet count, total cholesterol, triglycerides, high‐density lipoprotein cholesterol (HDL‐C), low‐density lipoprotein cholesterol (LDL‐C), serum creatinine, fasting glucose, serum potassium, and B‐type natriuretic peptide (BNP). All laboratory indicators were tested from emergency blood samples upon admission.

Strict data quality control was implemented in this study. All collected clinical indicators were double‐checked by professional clinicians to ensure data accuracy. For missing data handling, all enrolled patients had extremely low missing values for candidate variables, with an overall missing rate of less than 1.5% for all variables, and no variable had a missing rate higher than 5%. Missing data were identified as completely random missing (MCAR), with no systematic missing pattern associated with patient baseline condition, surgical complexity, or in‐hospital prognosis. Patients with massive key data missing had been excluded in advance according to the exclusion criteria.

### 2.4. Outcome Definition

The primary endpoint of this study was in‐hospital MACE, defined as the following composite events occurring from admission to discharge: cardiogenic shock; malignant arrhythmias (including sustained ventricular tachycardia, ventricular fibrillation, and high‐degree atrioventricular block); acute heart failure; reinfarction; and all‐cause death. Patients were divided into MACE and non‐MACE groups based on the occurrence of in‐hospital MACE.

### 2.5. Sample Size Calculation

According to empirical rules for prediction model studies, at least 10 positive events are required for each candidate predictor variable to ensure stable parameter estimation [14]. In this study, there were 260 cases in the MACE group, with 30 initial candidate variables. After LASSO screening, eight variables were finally included, corresponding to approximately 32.5 events per variable, fully meeting modeling requirements.

### 2.6. Statistical Analysis

All statistical analyses were performed using R software (Version 4.3.2, R Foundation for Statistical Computing, Vienna, Austria) and SPSS 26.0 software. Normality testing for continuous variables was performed using the Kolmogorov–Smirnov test. Normally distributed continuous variables were expressed as mean ± standard deviation (*x* ± *s*) with independent samples *t*‐test for group comparisons. Nonnormally distributed continuous variables were expressed as median (interquartile range) [M(Q_1_,Q_3_)] with Mann–Whitney *U* test for group comparisons. Categorical variables were expressed as frequency (percentage) [*n*(%)] with Pearson *χ* ^2^ test for group comparisons. Considering the extremely low and completely random missing data in this study, complete‐case analysis was strictly adopted for all statistical analyses. No multiple imputation was performed because the negligible missing proportion would not cause selection bias or affect the stability of model estimation, and complete‐case analysis could maximally retain the authenticity of original real‐world clinical data.

To ensure rigorous internal validation and avoid overfitting bias, a hybrid and robust internal validation strategy combining stratified random hold‐out validation and 1000 bootstrap resampling optimism correction was applied in this study. Considering the sufficient overall sample size and event number, all enrolled patients were divided into training set (*n* = 837) and independent validation set (*n* = 358) at a 7:3 stratified random ratio, which strictly guaranteed consistent MACE event proportions in the two datasets and enabled unbiased evaluation of model generalization on unseen data. Notably, the training set possessed adequate statistical power for model construction, with approximately 32.5 positive events per predictor, far exceeding the standard 10‐events‐per‐variable rule, which fully offset the statistical power loss caused by data partitioning.

For variable selection, a standardized two‐step screening strategy combining univariate logistic regression and LASSO regression was applied. Considering the large number of initial candidate variables, univariate logistic regression was first conducted in the training set with *p* < 0.05 as the preliminary screening threshold to exclude statistically insignificant noise variables and reduce model dimensionality. Notably, to avoid omitting clinically vital prognostic predictors caused by simple statistical preselection, variables with confirmed clinical predictive value for post‐PCI MACE in STEMI patients based on published guidelines and authoritative literature were retained for subsequent analysis regardless of univariate *p* values. After preliminary filtering, qualified candidate variables were imported into LASSO regression. Tenfold cross‐validation was used to determine the optimal penalty parameter *λ* (lambda.1se), and variables with nonzero regression coefficients were finally included in the model. LASSO regularization served as the decisive variable selection procedure, which eliminated multicollinearity, optimized variable combination, and prevented overfitting, overcoming the limitations of single univariate screening and traditional stepwise regression. Subsequently, LASSO regression was used for variable selection, with optimal penalty parameter *λ* determined through 10‐fold cross‐validation (selecting lambda.1se), finally including variables with nonzero regression coefficients. LASSO regression effectively reduces multicollinearity while achieving automatic variable selection, avoiding overfitting risks of traditional stepwise regression methods [15].

Variables selected by LASSO regression were included in the multivariate logistic regression model. Variance inflation factor (VIF) was tested for each variable, with VIF < 5 indicating no significant multicollinearity. Regression coefficients (*β* values) of the final model variables were used for model construction. Receiver operating characteristic (ROC) area under the curve (AUC) was used to evaluate model discrimination ability. AUC > 0.80 was considered to indicate good discrimination. The optimal cutoff value was determined based on the Youden index (Youden index = sensitivity + specificity − 1), with calculation of sensitivity, specificity, positive predictive value, and negative predictive value. The DeLong test was used to compare AUC differences between the combined model and individual predictors. To verify the incremental value of the novel model, we calculated GRACE and TIMI risk scores for all enrolled patients, plotted their ROC curves, and adopted the DeLong test to compare the discriminative performance between our model and the two classic clinical scoring systems. Model calibration was comprehensively evaluated using modern multi‐index quantitative strategies, rather than relying solely on the traditional Hosmer–Lemeshow (H‐L) test with low sensitivity and limitations. The evaluation system included Brier score (quantifying overall predictive error), calibration intercept (reflecting systematic prediction bias), calibration slope (reflecting model overfitting/underfitting degree), calibration curve visualization, and H‐L goodness‐of‐fit test. Brier score ranges from 0 to 1, with lower values indicating higher predictive accuracy; calibration intercept close to 0 and calibration slope close to 1 indicate optimal model calibration.

Omnibus test was applied for comprehensive testing of model coefficients (*p* < 0.05 indicates overall model significance). Decision curve analysis (DCA) was used to evaluate clinical net benefit of the model at different threshold probabilities, comparing the model with “treat all” and “treat none” extreme strategies. Model AUC, calibration, and DCA performance were evaluated in the independent validation set. Furthermore, 1000‐times bootstrap resampling was performed to calculate optimism‐corrected discrimination and calibration indicators, eliminating in‐sample overfitting bias and quantifying the true generalized performance of the prediction model. The optimism‐corrected AUC and corrected calibration slope were obtained to verify model stability and reliability. All statistical tests were two‐sided, with *p* < 0.05 considered statistically significant.

## 3. Results

### 3.1. Study Population and Baseline Characteristics

This study finally included 1195 STEMI patients meeting the criteria. Baseline characteristic comparisons between the two groups are shown in Table 1, with main findings as follows: MACE group patients were significantly older than the non‐MACE group [67 (58, 76) years versus 62 (52, 73), *p* < 0.001], with lower BMI [24.34 (22.63, 27.69) vs. 25.30 (22.99, 27.97), *p* = 0.031]. MACE group admission SBP [117 (94, 133) vs. 134 (119, 149), *p* < 0.001] and DBP [73 (62, 87.75) vs. 85 (75, 97), *p* < 0.001] were both significantly lower than the non‐MACE group. There was no significant difference in heart rate between groups (*p* = 0.763).MACE group Killip classification was significantly higher than the non‐MACE group [1 (1, 2) vs. 1 (1, 1), *p* < 0.001]. MACE group had lower proportions of males (67.3% vs. 74.0%, *p* = 0.032) and hypertension history (51.5% vs. 59.3%, *p* = 0.026) compared with the non‐MACE group. There were no significant differences between groups in hyperlipidemia (*p* = 0.651), diabetes (*p* = 0.207), or smoking history (*p* = 0.646). FMC time (*p* = 0.313) and D to W time (*p* = 0.300) showed no significant differences between groups, suggesting comparability in prehospital delays and in‐hospital reperfusion processes. MACE group intervention duration was significantly longer than the non‐MACE group [54 (43, 69.75) vs. 41 (32, 52), *p* < 0.001], and contrast volume was also significantly higher [105 (90, 128.75) vs. 85 (70, 100), *p* < 0.001]. Preprocedural TIMI grade (*p* < 0.001) and postprocedural TIMI grade (*p* < 0.001) both showed significant differences between groups. MACE group culprit vessel occlusion proportion was significantly higher than the non‐MACE group (88.8% vs. 72.1%, *p* < 0.001). Among culprit vessel locations, left anterior descending (*p* < 0.001) and right coronary artery (*p* < 0.001) showed statistical differences between groups. Gensini score showed statistical difference between groups (*p* = 0.032). There were no significant differences between groups in white blood cell count (*p* = 0.380), hemoglobin (*p* = 0.555), platelet count (*p* = 0.667), or glucose (*p* = 0.294). MACE group serum creatinine was significantly higher than the non‐MACE group [77.8 (64.35, 108.55) vs. 71.9 (59.50, 87.90), *p* < 0.001], and serum potassium was significantly lower [3.78 (3.39, 4.13) vs. 3.97 (3.64, 4.35), *p* < 0.001]. Among lipid profiles, only triglycerides showed statistical difference between groups [1.25 (0.82, 1.98) vs. 1.38 (0.94, 2.10), *p* = 0.013].

**Table 1:** Comparison of clinical data of the two groups of patients.

Variable	Non‐MACE (*n* = 935)	MACE (*n* = 260)	*p*
General clinical data			
Age	62 (52, 73)	67 (58, 76)	< 0.001
BMI	25.3 (22.99, 27.97)	24.34 (22.63, 27.69)	0.031
SBP	134 (119, 149)	117 (94, 133)	< 0.001
DBP	85 (75, 97)	73 (62, 87.75)	< 0.001
Heart rate	77 (65, 87)	74 (63, 90.75)	0.763
Killip class	1 (1, 1)	1 (1, 2)	< 0.001
Male	692 (74.00)	175 (67.30)	0.032
Hypertension	554 (59.30)	134 (51.50)	0.026
Hyperlipidemia	207 (22.10)	61 (23.50)	0.651
Diabetes	159 (17.00)	53 (20.40)	0.207
History of smoking	309 (33.00)	82 (31.50)	0.646
CAG‐PCI data			
FMC	248 (130, 470)	217.5 (119.25, 425)	0.313
D to W	72 (59, 85)	77 (57.25, 93)	0.300
Intervention time	41 (32, 52)	54 (43, 69.75)	< 0.001
Contrast agent dosage	85 (70, 100)	105(90, 128.75)	< 0.001
Pre‐PCI TIMI	0 (0, 1)	0 (0, 0)	< 0.001
Post‐PCI TIMI	3 (3, 3)	3 (3, 3)	< 0.001
Gensini score	56 (34, 80)	56 (34, 82)	0.032
Criminal vascular occlusion	674 (72.10)	231 (88.80)	< 0.001
Criminal vessel			
LM	9 (1.0)	4 (1.5)	0.650
LAD	555 (59.40)	97 (37.30)	< 0.001
LCX	84 (9.0)	22 (8.5)	0.793
RCA	287 (30.70)	137 (52.70)	< 0.001
Laboratory index			
WBC	10.6 (8.75, 13.02)	10.615 (8.69, 13.67)	0.380
Hemoglobin	140 (127, 152)	139 (124, 152.75)	0.555
Platelets	221 (185, 261)	223.5 (183.25, 259.75)	0.667
Total cholesterol	4.83 (4.08, 5.67)	4.755 (3.8225, 5.63)	0.126
Triglycerides	1.38 (0.94, 2.10)	1.25 (0.82, 1.98)	0.01
HDL‐C	1.21 (1, 1.43)	1.22 (0.97, 1.47)	0.660
LDL‐C	2.98 (2.34, 3.57)	2.89 (2.31, 3.67)	0.772
Cr	71.9 (59.50, 87.90)	77.8 (64.35, 108.55)	< 0.001
Glu	8.25 (7.09, 10.21)	8.56 (6.9725, 11.75)	0.294
Serum potassium	3.97 (3.64, 4.35)	3.78 (3.39, 4.13)	< 0.001
BNP	151.7 (75.44, 343.40)	166.235 (78.20, 412.01)	0.036

Abbreviations: BMI, body mass index; BNP, B‐type natriuretic peptide; Cr, serum creatinine; D to W, door to wire; DBP, diastolic blood pressure; FMC, first medical contact; Glu, fasting glucose; HDL‐C, high‐density lipoprotein cholesterol; LDL‐C, low‐density lipoprotein cholesterol; PCI, percutaneous coronary intervention; SBP, systolic blood pressure.

### 3.2. Univariate Logistic Regression Analysis

Univariate logistic regression analysis was performed in the training set (Table 2). Using *p* < 0.05 as the screening criterion, the following variables entered the candidate variable set: age (OR = 1.026, *p* < 0.001), SBP (OR = 0.970, *p* < 0.001), DBP (OR = 0.958, *p* < 0.001), Killip classification (OR = 3.256, *p* < 0.001), male gender (OR = 0.723, *p* = 0.033), hypertension (OR = 0.731, *p* = 0.026), intervention duration (OR = 1.036, *p* < 0.001), contrast volume (OR = 1.023, *p* < 0.001), preprocedural TIMI grade (OR = 0.663, *p* < 0.001), postprocedural TIMI grade (OR = 0.453, *p* < 0.001), Gensini score (OR = 1.007, *p* = 0.003), culprit vessel occlusion (OR = 3.085, *p* < 0.001), serum creatinine (OR = 1.011, *p* < 0.001), glucose (OR = 1.032, *p* = 0.049), serum potassium (OR = 0.520, *p* < 0.001), and BNP (OR = 1.013, *p* = 0.004), totaling 16 variables.

**Table 2:** Univariate logistic regression analysis of MACE.

Variables	Univariate logistic regression analysis		
	*β*	*p*	OR (95% CI)
Age	0.025	< 0.001	1.026 (1.014–1.037)
SBP	−0.03	< 0.001	0.97 (0.964–0.976)
DBP	−0.043	< 0.001	0.958 (0.949–0.967)
Killip class	1.181	< 0.001	3.256 (2.488–4.261)
Male	−0.324	0.033	0.723 (0.537–0.973)
Hypertension	−0.313	0.026	0.731 (0.555–0.964)
Intervention time	0.036	< 0.001	1.036 (1.029–1.044)
Contrast agent dosage	0.023	< 0.001	1.023 (1.019–1.028)
Pre‐PCI TIMI	−0.411	< 0.001	0.663 (0.56–0.785)
Post‐PCI TIMI	−0.792	< 0.001	0.453 (0.331–0.619)
Gensini score	0.007	0.003	1.007 (1.002–1.011)
Criminal vascular occlusion	1.126	< 0.001	3.085 (2.044–4.656)
Cr	0.011	< 0.001	1.011 (1.007–1.015)
Glu	0.032	0.049	1.032 (1–1.065)
Serum potassium	−0.654	< 0.001	0.52 (0.404–0.670)
BNP	0.012	0.004	1.013 (1.006–1.017)

Abbreviations: BNP, B‐type natriuretic peptide; Cr, serum creatinine; DBP, diastolic blood pressure; Glu, fasting glucose; SBP, systolic blood pressure.

### 3.3. LASSO Regression Variable Selection

The above 16 candidate variables were included in the LASSO regression model. Through 10‐fold cross‐validation, the optimal penalty parameter *λ* value was determined at lambda.1se (Figure 1A), with the corresponding variable coefficient path shown in Figure 1B. Finally, eight variables with nonzero regression coefficients were selected: SBP, Killip classification, intervention duration, contrast volume, postprocedural TIMI flow grade, culprit vessel occlusion, serum creatinine, and serum potassium. The regression coefficients for age, DBP, male gender, hypertension, preprocedural TIMI grade, Gensini score, glucose, and BNP were compressed to zero during LASSO regularization, suggesting these variables had limited independent predictive value for MACE after controlling for other factors.

**Figure 1:**
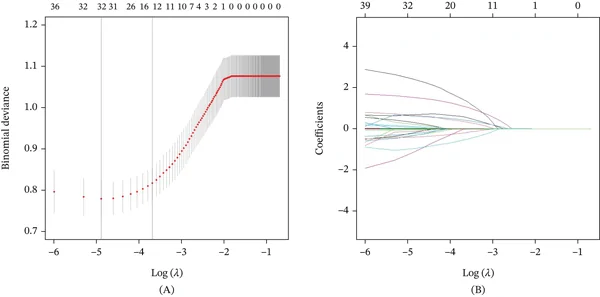
Screening for LASSO regression variables. Identify the optimal penalty parameter *λ* value (A), corresponding to the variable coefficient path diagram (B).

### 3.4. Multivariate Logistic Regression Model and Nomogram Model Construction

The eight variables selected by LASSO were included in the multivariate logistic regression model. All variables had VIF values < 5 (range 1.06–2.31), indicating no significant multicollinearity. Results showed that all eight variables were independent predictors of in‐hospital MACE (all *p* < 0.05), detailed in Table 3 and Figure 2. The regression coefficients and clinical interpretations for each predictor were as follows: admission SBP (*β* = −0.023, OR = 0.977, 95% CI: 0.970–0.984, *p* < 0.001): Each 1‐mmHg increase in SBP reduced MACE risk by 2.3%. Higher SBP levels at admission may reflect better cardiac pump function reserve. Killip classification (*β* = 0.870, OR = 2.387, 95% CI: 1.753–3.250, *p* < 0.001): Each 1‐grade increase in Killip classification increased MACE risk by 138.7%, representing one of the strongest independent predictors. Intervention duration (*β* = 0.012, OR = 1.013, 95% CI: 1.003–1.023, *p* = 0.013): Each 1‐min increase in procedure time increased MACE risk by 1.3%. Contrast volume (*β* = 0.017, OR = 1.017, 95% CI: 1.011–1.023, *p* < 0.001): Each 1‐mL increase in contrast agent increased MACE risk by 1.7%. Postprocedural TIMI flow grade (*β* = −0.578, OR = 0.561, 95% CI: 0.378–0.832, *p* = 0.004): Each 1‐grade increase in postprocedural TIMI grade reduced MACE risk by 43.9%. Culprit vessel occlusion (*β* = 0.889, OR = 2.433, 95% CI: 1.499–3.948, *p* < 0.001): Patients with complete culprit vessel occlusion had 143.3% increased MACE risk. Serum creatinine (*β* = 0.012, OR = 1.012, 95% CI: 1.007–1.017, *p* < 0.001): Each 1‐*μ*mol/L increase in creatinine increased MACE risk by 1.2%. Serum potassium (*β* = −0.573, OR = 0.564, 95% CI: 0.422–0.753, *p* < 0.001): Each 1‐mmol/L increase in serum potassium reduced MACE risk by 43.6%. The final prediction model formula was: logit(p) = −0.023 × SBP + 0.87 × Killip + 0.012 × intervention duration + 0.017 × contrast volume − 0.578 × post − TIMI grade + 0.889 × culprit vessel occlusion + 0.012 × creatinine − 0.573 × potassium + 0.494, where culprit vessel occlusion is a binary variable (occlusion = 1, nonocclusion = 0), and other variables are continuous. A nomogram model was also constructed (Figure 3).

**Table 3:** Multivariate logistic regression analysis of MACE (include SBP/Killip class/intervention time/contrast agent dosage/post‐PCI TIMI/criminal vascular occlusion/Cr and serum potassium only).

	*β*	S·E	Wals	*p*	OR (95% CI)
SBP	−0.023	0.004	39.673	< 0.001	0.977 (0.970–0.984)
Killip class	0.870	0.157	30.542	< 0.001	2.387 (1.753–3.250)
Intervention time	0.012	0.005	6.221	0.013	1.013 (1.003–1.023)
Contrast agent dosage	0.017	0.003	29.030	< 0.001	1.017 (1.011–1.023)
Post‐PCI TIMI	−0.578	0.201	8.253	0.004	0.561 (0.378–0.832)
Criminal vascular occlusion	0.889	0.247	12.948	< 0.001	2.433 (1.499–3.948)
Cr	0.012	0.002	23.519	< 0.001	1.012 (1.007–1.017)
Serum potassium	−0.573	0.147	15.074	< 0.001	0.564 (0.422–0.753)
Constant	0.494	1.080	0.209	0.648	1.638

Abbreviations: Cr, serum creatinine; SBP, systolic blood pressure.

**Figure 2:**
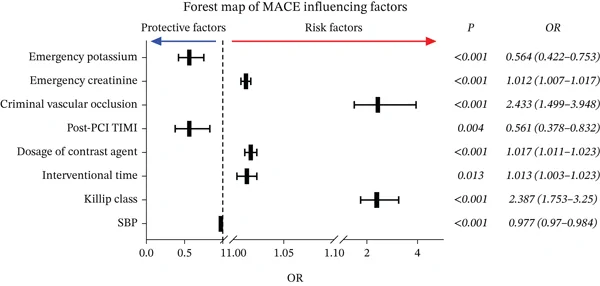
In order to show the OR value of each indicator, the *x*‐axis of the graph is divided into three segments, namely, 0–1, 1.00–1.10, and 1.10–4. SBP, systolic blood pressure; PCI, percutaneous coronary intervention.

**Figure 3:**
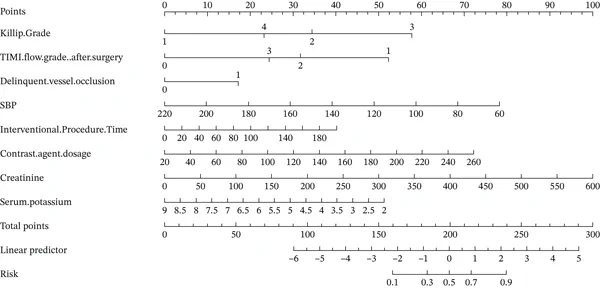
Nomogram model.

### 3.5. Model Evaluation

#### 3.5.1. Discrimination Assessment

The combined prediction model AUC in the training set was 0.84 (95% CI: 0.80–0.87). Based on the Youden index, the optimal cutoff value was determined to be 0.205 (corresponding to logit(p) = −1.355), with sensitivity of 77.7% and specificity of 78.2% (Table 4). The combined prediction model AUC was significantly superior to individual predictors (Table 4). The DeLong test showed that differences between the combined model AUC and individual factor AUCs were all statistically significant (all *p* < 0.05). The model AUC in the validation set was 0.84 (95% CI: 0.79–0.89) (Figure 4A), suggesting good generalizability. After 1000 bootstrap resampling correction, the optimism‐corrected AUC of the model was 0.835 (95% CI: 0.786–0.882) (Figure 4B), with negligible optimism (*Δ*AUC = 0.005), indicating minimal overfitting and excellent stable discrimination ability of the constructed model. Head‐to‐head discriminative comparison with traditional risk scores showed that the GRACE score yielded an AUC of 0.763 in the training set and 0.751 in the validation set, whereas the TIMI score yielded an AUC of 0.748 in the training set and 0.736 in the validation set. All AUC values of GRACE and TIMI scores were below 0.8, indicating only moderate discriminative ability for post‐PCI in‐hospital MACE prediction. The DeLong test results confirmed that our novel model exhibited statistically significantly higher discriminative accuracy than both GRACE and TIMI scores in both training and validation cohorts (all *p* < 0.05).

**Table 4:** Predictive value of prediction model/SBP/Killip class/intervention time/contrast agent dosage/post‐PCI TIMI/criminal vascular occlusion/Cr and serum potassium.

	Cutoff	AUC	S·E	Sensitivity	Specificity	*p*	95% CI
Prediction model	0.205	0.842	0.014	0.777	0.782	< 0.001	0.814–0.871
SBP	123.500	0.693	0.020	0.674	0.635	< 0.001	0.654–0.732
Killip class	1.500	0.623	0.022	0.288	0.954	< 0.001	0.581–0.665
Interventional time	47.500	0.714	0.018	0.654	0.673	< 0.001	0.679–0.748
Dosage of contrast agent	87.500	0.742	0.016	0.862	0.555	< 0.001	0.711–0.774
Post‐PCI TIMI	2.500	0.548	0.021	0.957	0.138	0.017	0.410–0.493
Criminal vascular occlusion	0.500	0.584	0.019	0.888	0.279	< 0.001	0.547–0.621
Cr	107.950	0.591	0.021	0.262	0.888	< 0.001	0.551–0.631
Serum potassium	4.005	0.608	0.020	0.481	0.692	< 0.001	0.568–0.648

Abbreviations: Cr, serum creatinine; SBP, systolic blood pressure.

**Figure 4:**
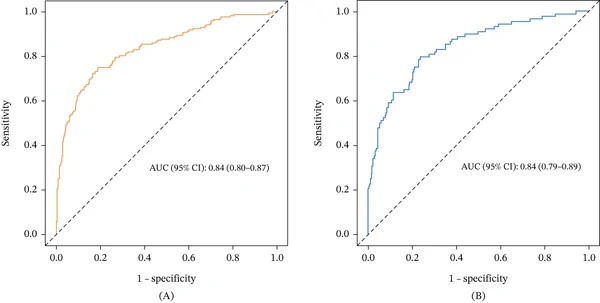
ROC curve analysis of predictive models. (A) Training set. (B) Validation set.

#### 3.5.2. Calibration Assessment

Omnibus test showed the model coefficients were statistically significant overall (*χ* ^2^ = 341.773, *p* < 0.001). Quantitative calibration indicators showed that the training set Brier score was 0.128, calibration intercept was 0.042, and calibration slope was 0.991; the validation set Brier score was 0.135, calibration intercept was 0.051, and calibration slope was 0.985. All Brier scores were far lower than 0.25, indicating excellent overall predictive accuracy; the intercepts were close to 0 and slopes were close to 1.0, demonstrating no systematic prediction bias and no obvious overfitting or underfitting.

H‐L goodness‐of‐fit test result was *χ* ^2^ = 5.449, *p* = 0.709 (> 0.05), indicating good consistency between model predicted probabilities and actual MACE incidence rates. The optimal threshold for combined predictors was calculated as 0.205 using the Youden index, yielding an optimal logit(p) diagnostic value of −1.355. When logit(p) > −1.355, MACE occurrence can be predicted. Training set calibration curves showed good fit between predicted and observed probabilities, with calibration slope close to 1.0 (Figure 5A). Validation set calibration curves also demonstrated good calibration (Figure 5B). Bootstrap‐corrected calibration slope was 0.987, which was close to 1.0, further confirming that the model has no systematic prediction deviation and presents excellent and stable calibration performance after eliminating internal optimism bias.

**Figure 5:**
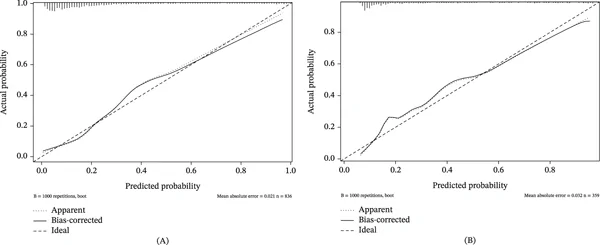
Calibration curve analysis of the prediction model. (A) Training set. (B) Validation set.

#### 3.5.3. Clinical Utility Assessment

DCA (Figure 6A,B) showed that within the threshold probability range of approximately 5%–80%, the combined prediction model′s net benefit was consistently higher than both “treat all” and “treat none” extreme strategies, indicating practical application value in clinical practice. Within the clinically common decision interval of 10%–30% threshold probability, the model′s net benefit advantage was particularly significant, suggesting good applicability in assisting clinicians with individualized management strategies.

**Figure 6:**
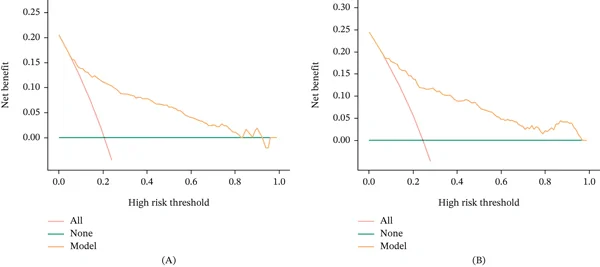
Decision curve analysis of predictive models. (A) Training set. (B) Validation set.

### 3.6. Model Application Example

To facilitate postoperative clinical risk assessment and practical application, we demonstrate with an example patient: A patient with admission SBP = 96 mmHg, Killip classification = 1, intervention duration = 47 min, contrast volume = 80 mL, postprocedural TIMI grade = 3, culprit vessel occlusion ( = 1), serum creatinine = 89.6 *μ*mol/L, and serum potassium = 4.04 mmol/L. All indicator data were completely obtained after the completion of PCI surgery. Calculation using the model: logit(*p*) = −0.023 × 96 + 0.87 × 1 + 0.012 × 47 + 0.017 × 80 + −0.578 × 3 + 0.889 × 1 + 0.012 × 89.6 + −0.573 × 4.04 + 0.494 = −1.005 > cutoff value −1.355. The model predicts this patient belongs to the MACE high‐risk population after PCI, recommending enhanced postoperative hemodynamic monitoring, optimized volume management, and targeted preventive interventions during hospitalization.

## 4. Discussion

This study, based on single‐center clinical data from the Chinese chest pain center system, systematically integrated admission baseline characteristics, intraoperative interventional parameters, and laboratory indicators, and constructed a targeted post‐PCI in‐hospital MACE prediction model for STEMI patients containing eight clinically accessible indicators via LASSO variable selection combined with multivariate logistic regression. Different from traditional preoperative risk scoring systems that only rely on baseline data, this model incorporates key intraoperative procedural parameters reflecting surgical complexity and myocardial reperfusion status, making up for the deficiency of existing tools in postoperative risk assessment. Head‐to‐head discriminative comparison verified that our novel model exhibits significantly superior predictive accuracy compared with classic GRACE and TIMI risk scores, which fully demonstrates the prominent incremental clinical value of integrating multidimensional perioperative indicators. The model achieved AUC of 0.84 in training set and 0.826 in validation set, demonstrating good discrimination, calibration, and clinical utility for postprocedural risk stratification. The in‐hospital MACE incidence was 21.8% (260/1,195), consistent with previous studies. Huang et al. [9] reported STEMI patient in‐hospital MACE rates of approximately 18.6%–23.4% based on Chinese chest pain center data, and Wu et al. [8] showed similar levels. The high in‐hospital MACE incidence further highlights the clinical urgency for effective prediction tools.

GRACE and TIMI scores are the most commonly used risk stratification tools for STEMI patients in clinical practice. However, consistent with our comparative results, these traditional scoring systems have inherent limitations in postoperative risk prediction for Chinese STEMI patients undergoing emergency PCI. Primarily derived from Western populations, they show suboptimal applicability to Chinese patient cohorts. More importantly, both tools only include preoperative baseline variables and ignore intraoperative procedural risk factors closely associated with postoperative MACE, such as operation duration and contrast dosage. In our cohort, both GRACE and TIMI scores only achieved moderate discriminative performance with AUC values below 0.8, which was significantly inferior to our newly developed model. These findings indicate that traditional preoperative scoring systems cannot fully capture the real perioperative risk and postoperative prognosis of STEMI patients after PCI.

The core innovation and incremental advantage of our model lies in the combination of preoperative baseline indicators, intraoperative interventional parameters and laboratory indexes. The four intraoperative indicators (intervention duration, contrast volume, postprocedural TIMI flow grade, culprit vessel occlusion) introduced in this model can dynamically reflect surgical complexity, intraoperative risk exposure and myocardial reperfusion quality, which are critical prognostic factors missing in GRACE and TIMI scoring systems. The significant improvement in discriminative ability confirms that integrating intraoperative perioperative information can effectively compensate for the defects of traditional risk models and achieve more accurate postoperative risk stratification.

Accumulating emerging evidence has demonstrated that inflammatory activation and endothelial dysfunction serve as core pathological mechanisms driving myocardial injury, microcirculatory disturbance, and subsequent adverse cardiovascular events after STEMI reperfusion, providing incremental prognostic value for post‐PCI MACE prediction beyond conventional clinical and procedural variables [15, 16]. As reported in recent high‐quality studies, multiple inflammation‐related biomarkers (e.g., high‐sensitivity C‐reactive protein, neutrophil‐to‐lymphocyte ratio) and endothelial dysfunction indicators can effectively reflect the severity of systemic inflammatory response and coronary microvascular impairment in STEMI patients, which are strongly associated with increased risks of malignant arrhythmia, acute heart failure and in‐hospital death after primary PCI [15, 16].

Specifically, a recent prospective cohort study confirmed that endothelial dysfunction biomarkers independently predict impaired myocardial reperfusion and short‐term adverse outcomes in STEMI patients undergoing PCI, providing unique prognostic information complementary to traditional anatomical and procedural indicators [15]. Another latest study further verified that systemic inflammatory biomarkers could effectively stratify in‐hospital cardiovascular risk and optimize prognostic evaluation for STEMI populations, even in patients with successful coronary recanalization [16]. These emerging biomarkers reflect the underlying molecular and inflammatory pathological changes of myocardial ischemia‐reperfusion injury, which cannot be fully captured by the macroscopic clinical, procedural, and routine laboratory variables adopted in our current model.

Nevertheless, our established eight‐variable model possesses distinct clinical practicability and accessibility compared with emerging inflammatory and endothelial biomarkers. All indicators included in our model are routine, real‐time, and universally detectable clinical parameters available in primary and tertiary hospitals, without additional expensive detection costs or specialized testing equipment, which ensures excellent clinical popularization and application value. In contrast, most novel inflammatory and endothelial dysfunction biomarkers are not included in routine emergency testing panels of chest pain centers, limiting their real‐time clinical application for immediate postoperative risk stratification. Moreover, the current model integrates intraoperative procedural characteristics that directly reflect surgical complexity and reperfusion quality, which are superior to single inflammatory biomarkers in reflecting comprehensive perioperative risk.

Notably, we fully acknowledge the limitations of our model in lacking inflammatory and endothelial‐related biomarkers. These emerging indicators can reflect the microscopic inflammatory and vascular endothelial pathological state of STEMI patients, representing a crucial direction for further model optimization. Future research will incorporate validated inflammatory and endothelial dysfunction biomarkers to construct a multidimensional prediction model covering macroscopic perioperative clinical indicators and microscopic molecular biomarkers, so as to further improve the accuracy and comprehensiveness of postoperative risk stratification.

Notably, consistent with the reviewer′s valuable suggestion, we cautiously emphasize that intervention duration and contrast volume included in the final model are not independent biological risk factors for MACE, but objective surrogate markers reflecting intraoperative procedural complexity and lesion severity. Their significant positive correlation with in‐hospital MACE is essentially a statistical reflection of high‐risk pathological and anatomical features of STEMI, rather than a direct causal biological relationship.

Prolonged intervention duration typically corresponds to complex clinical scenarios, including severe coronary calcification, tortuous vessels, massive thrombus burden, multivessel lesions, and difficult culprit vessel recanalization. Similarly, increased contrast volume is usually attributed to repeated angiography, difficult lesion positioning, and multiple intraoperative adjustments during complex operations. These complex lesion characteristics and difficult operative conditions are the fundamental drivers of poor postoperative prognosis, whereas procedural duration and contrast dosage are only external phenotypic manifestations of high surgical complexity.

In addition, this study acknowledges the potential existence of confounding by indication and residual confounding regarding the two intraoperative procedural indicators. Confounding by indication is mainly reflected in that patients with more severe baseline cardiac function, more serious coronary lesions and higher preoperative risk are more likely to undergo prolonged and complex PCI procedures with increased contrast usage; therefore, the elevated MACE risk corresponding to longer operation time and higher contrast volume may be partially attributed to the inherent high‐risk baseline status of patients rather than the procedural parameters themselves.

Moreover, potential residual confounding factors that cannot be fully adjusted may also affect the association between procedural parameters and MACE, including intraoperative hemodynamic fluctuation, unmeasured microcirculation dysfunction, intraoperative thrombus shedding, and subtle differences in interventional operator experience. Although we adopted strict variable screening, multivariate regression adjustment and standardized quality control to minimize confounding bias, complete elimination of residual confounding is difficult in real‐world retrospective clinical research.

Therefore, the prognostic value of intervention duration and contrast volume should be interpreted prudently. We do not consider these two indicators as independent causal risk factors for MACE. Instead, they serve as convenient, clinically accessible intraoperative surrogate variables that can effectively reflect the overall perioperative risk level of STEMI patients. Their inclusion significantly improves the model′s postoperative risk stratification efficiency, enabling the model to capture intraoperative risk information that cannot be reflected by traditional preoperative scoring systems.

In terms of model validation methodology, this study adopted a rigorous hybrid internal validation strategy to compensate for the limitations of single random data splitting. Traditional simple hold‐out validation may reduce effective sample size and fail to quantify model overfitting optimism, whereas single bootstrap validation lacks objective evaluation on independent unseen data. In this study, independent 7:3 stratified hold‐out validation ensured unbiased external internal verification of model generalization capability, and 1000 bootstrap resampling further calculated optimism‐corrected AUC and calibration indicators to eliminate in‐sample overfitting bias. The corrected AUC was 0.835 with tiny optimism deviation, and the corrected calibration slope was close to 1.0, fully proving that the model possesses stable discrimination and calibration without obvious overfitting. This dual validation strategy complies with the latest TRIPOD prediction model reporting guidelines and makes our model evaluation results more credible and reproducible.

This study found admission SBP to be an independent protective factor for MACE (OR = 0.977), with each 1‐mmHg SBP increase reducing MACE risk by 2.3%. This finding is consistent with multiple previous studies [9, 17, 18]. Higher SBP levels at admission reflect better cardiac pump function reserve to some extent, whereas hypotensive states suggest reduced cardiac output and hemodynamic instability, potentially exacerbating the vicious cycle of myocardial ischemia‐hypoxia. Pei et al. [17] demonstrated a significant negative correlation between admission SBP and MACE incidence, with increased coronary perfusion pressure potentially reducing thrombotic extension risk as SBP increases. Hu et al. [18] confirmed associations between admission blood pressure and spontaneous reperfusion and long‐term prognosis in STEMI patients through multicenter observational studies. Notably, although DBP showed significant differences between groups, it was compressed to zero during LASSO regularization and did not enter the final model. This may be related to high collinearity between systolic and diastolic pressures, with DBP′s independent predictive value weakened after adjusting for SBP. Liu et al. [13] indicated that low DBP (< 50 mmHg) is an independent predictor of long‐term mortality after PCI, but their study population had relatively well‐controlled SBP, differing from our STEMI patients in acute pathological states with generally low SBP. Therefore, in acute STEMI management, admission SBP may be a more predictive hemodynamic indicator than DBP.

Killip classification was one of the strongest independent predictors in our model (OR = 2.387), with each 1‐grade increase increasing MACE risk by 138.7%. As the most classic clinical tool for acute myocardial infarction cardiac function assessment, Killip classification rapidly reflects heart failure severity and correlates closely with infarct size [19–21]. Our results are highly consistent with clinical experience. Yang et al. [21] showed Killip classification′s prominence in in‐hospital MACE prediction using machine learning models based on Chinese myocardial infarction registry data. Jeon et al. [22] found significantly increased thrombus aspiration failure rates during emergency PCI in STEMI patients with Killip ≥ III, possibly related to coronary hemodynamic instability caused by high Killip grades. Notably, Killip classification primarily reflects left heart function status and has certain limitations in assessing right ventricular infarction‐related cardiac function. Future studies could explore integrating left and right heart function assessment indicators to improve predictive accuracy.

Intervention duration was an independent risk factor for MACE (OR = 1.013), with each 1‐min increase in procedure time increasing MACE risk by 1.3%. Notably, in the Chinese chest pain center quality control system, FMC time and D to W time are core quality control indicators [23], whereas intervention duration (from catheter sheath insertion to procedure completion) has not been included in key monitoring categories. In this study, FMC time and D to W time showed no significant differences between groups, with D to W time basically controlled within 90 min, meeting guideline recommendations. Koh et al. [24] also suggested that further shortening D to W time has limited benefit for patient outcomes when D to W < 90 min. However, prolonged procedure time may reflect surgical complexity (such as multivessel disease, severe calcification, large thrombus burden), whereas longer procedure time means prolonged myocardial ischemia duration, increased contrast exposure, and cumulative intravascular manipulation damage. Therefore, minimizing intervention duration while ensuring surgical quality and treating only the culprit vessel has positive significance for reducing in‐hospital MACE incidence.

Contrast volume was an independent risk factor for MACE (OR = 1.017), with each 1‐mL increase increasing MACE risk by 1.7%. Previous studies mainly focused on associations between contrast agents and acute kidney injury [25, 26]. Qiu et al. [27] developed a nomogram model predicting contrast‐induced acute kidney injury after PCI in STEMI patients. This study further revealed independent associations between contrast volume and in‐hospital MACE. Possible mechanisms include: Larger contrast volumes often reflect surgical complexity and need for repeated angiography; contrast agents can increase blood viscosity, obstructing myocardial microcirculation; contrast nephrotoxicity can induce acute kidney injury, subsequently increasing cardiac burden through cardiorenal interactions [25, 26]. Our results suggest minimizing contrast volume during emergency PCI, using low‐osmolar or iso‐osmolar contrast agents, and strengthening postprocedural fluid management.

Postprocedural TIMI flow grade was an independent protective factor for MACE (OR = 0.561), with each 1‐grade increase reducing MACE risk by 43.9%. TIMI flow grade is the classic indicator for assessing coronary reperfusion effectiveness [13], with higher postprocedural TIMI grades reflecting effective culprit vessel opening and coronary antegrade flow restoration. This study′s findings are consistent with San Román Arispe et al. [28]. However, it is important to note that post‐PCI TIMI 3 flow does not equate to complete myocardial‐level reperfusion. Xie et al. [29] showed that at least half of patients achieving TIMI 3 flow had incomplete coronary microvascular perfusion recovery, with this “no‐reflow” or “slow‐flow” phenomenon significantly associated with increased mortality [30]. Future studies could consider incorporating myocardial blush grade or microcirculatory resistance index to further improve model assessment of myocardial‐level reperfusion status.

Notably, although preprocedural TIMI grade showed statistical differences between groups, it was excluded during LASSO screening, suggesting limited independent predictive value after adjusting for culprit vessel occlusion, postprocedural TIMI grade, and other variables. Complete culprit vessel occlusion was an independent risk factor for MACE (OR = 2.433), increasing MACE risk by 143.3%. Complete culprit vessel occlusion means complete interruption of antegrade flow in the infarct‐related artery, with myocardium in severe ischemic state, often resulting in larger infarct size and more severe myocardial damage. The MACE group′s culprit vessel occlusion proportion (88.8%) was significantly higher than the non‐MACE group (72.1%), fully demonstrating complete occlusion′s adverse prognostic impact. Additionally, completely occluded vessels are more difficult to open during PCI, potentially requiring more procedure time and contrast agent, creating multiple adverse factor overlay effects.

Serum creatinine was an independent risk factor for MACE (OR = 1.012), with each 1‐*μ*mol/L increase increasing MACE risk by 1.2%. Although the risk increase per unit change is small, considering the large variation range of creatinine in clinical practice, its cumulative effect should not be ignored. Our results are consistent with previous literature [20, 31]. Associations between renal dysfunction and MACE may be realized through the following pathways: Renal dysfunction can cause volume overload, increasing cardiac preload; uremic toxin accumulation can damage endothelial function, promoting atherosclerosis progression; Savic et al. [31] found renal dysfunction to be an independent predictor of post‐PCI slow‐flow/no‐reflow phenomena, whereas slow‐flow/no‐reflow is a powerful predictor of short‐term and long‐term mortality. This study used admission emergency creatinine levels, with the advantage of pre‐PCI availability, facilitating preprocedural risk stratification.

Serum potassium was an independent protective factor for MACE (OR = 0.564), with each 1‐mmol/L increase reducing MACE risk by 43.6%. Hypokalemia is a common electrolyte disorder in STEMI patients, with associations with MACE mainly reflected in malignant arrhythmias. Hypokalemia can cause myocardial cell membrane potential instability, reduce ventricular fibrillation threshold, and increase occurrence risk of sustained ventricular tachycardia and ventricular fibrillation [32–34]. Wang et al. [32] included serum potassium levels as an important predictor in their malignant ventricular arrhythmia prediction nomogram model. Sun et al. [34] confirmed hypokalemia′s predictive value in their ventricular arrhythmia risk scoring system. Our results suggest high attention to serum potassium monitoring and management during acute STEMI, with early potassium supplementation for hypokalemic patients to maintain serum potassium within normal ranges.

The clinical positioning of this model needs to be clearly distinguished from traditional preoperative prediction tools: The core variables of intervention duration, contrast volume, and postprocedural TIMI flow grade can only be obtained after PCI completion, so this model cannot be used for preoperative risk prediction. Its core clinical application scenario is rapid and accurate in‐hospital MACE risk stratification for patients immediately after emergency PCI. It compensates for the defect that traditional preoperative scoring systems cannot reflect intraoperative surgical risk and reperfusion status and can guide clinicians to implement targeted intensified monitoring and individualized intervention for high‐risk patients in the early postoperative period, so as to reduce the in‐hospital MACE rate.

This study has the following limitations: (1) This was a single‐center retrospective design with potential selection and information bias, requiring cautious evaluation of result generalizability. Future multicenter prospective studies are needed for external validation to confirm model applicability across different populations and medical institutions. (2) As emphasized above, intervention duration and contrast volume are surrogate markers of procedural complexity rather than independent biological risk factors, and the associations between these two indicators and MACE may be affected by confounding by indication and residual confounding. Further prospective studies with stricter confounding control and detailed intraoperative lesion evaluation are required to further verify their predictive value and causal relationship. (3) This study only targeted STEMI patients receiving emergency PCI, not covering non–ST‐segment elevation myocardial infarction (NSTEMI) patients, with certain limitations in model applicability scope. (4) This study did not include certain potentially valuable biomarkers (such as high‐sensitivity troponin peak, high‐sensitivity C‐reactive protein, and glycated hemoglobin) or echocardiographic indicators (such as left ventricular ejection fraction). Future studies could consider integrating more dimensional predictive indicators to further optimize model performance. (5) Although training/validation set division and bootstrap resampling were used for internal validation, external validation has not been performed, with model generalizability requiring further confirmation from multicenter data. (6) Limited by the inclusion of intraoperative postprocedural indicators, the model can only be applied to postoperative risk assessment rather than preoperative early prediction. Subsequent research can further screen pure preoperative baseline indicators to construct a complementary preoperative prediction model, so as to realize whole‐process perioperative risk stratification for STEMI patients undergoing PCI.

In conclusion, based on clinical data from 1195 STEMI patients, this study used LASSO regression combined with multivariate logistic regression to construct a post‐PCI in‐hospital MACE prediction model including eight clinically accessible indicators: admission SBP, Killip classification, intervention duration, contrast volume, postprocedural TIMI flow grade, culprit vessel occlusion, serum creatinine, and serum potassium. AUC comparison and the DeLong test confirmed that the novel model possesses significantly better discriminative ability than conventional GRACE and TIMI risk scores, with obvious incremental predictive value for postoperative risk assessment. The model demonstrated good discrimination (training set AUC = 0.84, validation set AUC = 0.826), reliable calibration (H‐L test *p* = 0.709), and stable performance after bootstrap optimism correction and internal validation. This model can accurately stratify postoperative in‐hospital MACE risk of STEMI patients after emergency PCI, assist clinicians in identifying high‐risk populations in the early postoperative period, and optimize postoperative perioperative monitoring and individualized treatment strategies, with superior clinical application prospects than conventional risk scoring systems.

## Author Contributions

Jun Li and Na Wang designed the study. Geng Pan extracted the data. Na Wang and Mei Wu analyzed the data. Jun Li drafted the manuscript, which was revised by Na Wang and Mei Wu. Jun Li and Geng Pan have contributed to the work equally and should be regarded as cofirst authors.

## Funding

No funding was received for this manuscript.

## Disclosure

All authors approved the final version of the manuscript.

## Ethics Statement

The study was approved by the Ethics Committee of the Luan People′s Hospital, and the study protocol conformed to the principles of the Declaration of Helsinki.

## Conflicts of Interest

The authors declare no conflicts of interest.

## Data Availability

The data that support the findings of this study are available from the corresponding authors upon reasonable request.
